# Epidemiologic analysis of families with isolated anorectal malformations suggests high prevalence of autosomal dominant inheritance

**DOI:** 10.1186/s13023-017-0729-7

**Published:** 2017-12-13

**Authors:** Gabriel C. Dworschak, Nadine Zwink, Eberhard Schmiedeke, Kiarasch Mortazawi, Stefanie Märzheuser, Konrad Reinshagen, Johannes Leonhardt, Barbara Gómez, Patrick Volk, Anke Rißmann, Ekkehart Jenetzky, Heiko Reutter

**Affiliations:** 1 Institute of Human Genetics, University of Bonn, Bonn, Germany; 2 Department of Pediatrics, Children’s Hospital, University of Bonn, Sigmund-Freud-Str. 25, D-53127 Bonn, Germany; 3 Division of Clinical Epidemiology and Aging Research, German Cancer Research Center, Heidelberg, Germany; 4 Department of Child and Adolescent Psychiatry, University Medical Center of the Johannes Gutenberg University Mainz, Mainz, Germany; 5 Department of Pediatric Surgery and Urology, Center for Child and Adolescent Health, Hospital Bremen-Mitte, Bremen, Germany; 6 Department of Pediatric Surgery, Municipal Hospital, Karlsruhe, Germany; 7 Department of Pediatric Surgery, Campus Virchow Clinic, Charité University Hospital Berlin, Berlin, Germany; 8 Department of Pediatric Surgery, University Medical Center Hamburg-Eppendorf/Altona Children’s Hospital, Hamburg, Germany; 9 Department of Pediatric Surgery, St. Bernward-Hospital, Hildesheim, Germany; 10 Department of Pediatric Surgery, Children’s and Youth Hospital “Auf der Bult”, Hannover, Germany; 11 Department of Surgery, University of Heidelberg, Heidelberg, Germany; 12 Malformation Monitoring Centre Saxony-Anhalt, Otto-von-Guericke University, Magdeburg, Germany; 13 Child Center Maulbronn GmbH, Hospital for Pediatric Neurology and Social Pediatrics, Maulbronn, Germany; 14 Department of Neonatology and Pediatric Intensive Care, Children’s Hospital, University of Bonn, Bonn, Germany

**Keywords:** Anorectal malformation, ARM, Inheritance, Recurrence risk, Genetic counseling

## Abstract

**Background:**

Anorectal malformations (ARM) are rare abnormalities that occur in approximately 1 in 3000 live births with around 40% of patients presenting with isolated forms. Multiple familial cases reported, suggest underlying genetic factors that remain largely unknown. The recurrence in relatives is considered rare, however transmission rates of ARM by affected parents have never been determined before. The inheritance pattern of ARM was investigated in our database of patients with isolated ARM.

**Results:**

Within our cohort of 327 patients with isolated ARM we identified eight adult patients from eight families who had in total 16 children with their healthy spouse. Of these ten had ARM, resulting in a recurrence risk of approximately one in two live births (10 of 16; 62%). From 226 families with 459 siblings we found two affected siblings in five families. Hence, the recurrence risk of ARM among siblings is approximately one in 92 live births (5 of 459; 1.0%).

**Conclusions:**

Comparing the observed recurrence risk in our cohort with the prevalence in the general population, we see a 1500-fold increase in recurrence risk for offspring and a 32-fold increase if a sibling is affected. The recurrence risk of approximately 62% indicates an autosomal dominant mode of inheritance. Reliable figures on recurrence of ARM are becoming increasingly important since improved surgical techniques are able to maintain sexual function resulting in more offspring of patients with ARM. These data allow more precise counseling of families with ARM and support the need for genetic studies.

**Electronic supplementary material:**

The online version of this article (10.1186/s13023-017-0729-7) contains supplementary material, which is available to authorized users.

## Background

Anorectal malformations (ARM) comprise a broad spectrum, ranging from mild anal anomalies to complex cloacal malformations. The estimated birth prevalence amount to 1 in 3000 live births, with a male to female ratio of 1.7 [1–4]. Around 60% of ARM occur within the context of defined genetic syndromes or complex multiple congenital anomalies or in association with chromosomal aberrations [4, 5]. The remaining 40% are isolated ARM with its etiology remaining largely unknown.

Although we previously identified 59 ARM families from the literature with at least two affected members [6], the condition is usually sporadic, with low risk of recurrence among first degree relatives [4]. In the first report on the risk of recurrence from Anderson and Reed the likelihood for recurrence of ARM in a sibling has been depicted with 1 in 100 (1%) [7]. However, this figure is based on surveillance of not further described patients from unquoted reports. In 2007 Falcone et al. calculated the risk of recurrence in a large cohort of 1606 patients with ARM with 1.4% without specifying between risk for siblings or risk for offspring [8]. Here we present inheritance data on a cohort of 619 ARM patients and demonstrate evidence for monogenic inheritance in at least a subset of patients.

## Methods

In 2009 the last two authors initiated a nationwide German study of the genetic causes of urogenital and anorectal malformations (CURE-Net, Network for Congenital Uro-REctal malformations). Patients and their families have been contacted through the German self-help organization for patients with anorectal malformations (SoMA e.V.) and various German pediatric surgical departments. The CURE-Network comprises 23 pediatric surgical departments in Germany. Recruiting physicians are experienced in the field of congenital anomalies and are encouraged to contribute any case, regardless of sporadic or multiple occurrence of the ARM. Cases underwent a clinical investigation and ARM was classified according to the Krickenbeck-Classification [9]. Despite major efforts to collect comprehensive data in Germany, it cannot be claimed that the cohort represents population-based data.

Data on multiple affected family members was compiled using a standardized questionnaire. Information regarding the type of ARM, associated malformations and family history were evaluated. In order to avoid overestimation of recurrence risk and to exclude any form of non-isolated ARM, strict criteria have been applied to identify isolated cases for further analysis: If any malformation outside the anorectal developmental field was noted, the case was flagged as “non-isolated” and excluded. Anomalies within the anorectal developmental field include anomalies of the upper and lower urinary tract as well as the genitalia and the os sacrum. In contrast, patients presenting with any of the following features were defined as non-isolated cases and excluded from the analysis: Chromosomal or single gene disorders; any defined clinical syndrome; congenital malformations of the esophagus, the heart, the limbs, mental retardation; and facial anomalies (e.g., orofacial clefts or hemifacial macrosomia). If it was uncertain whether the patient should be classified isolated or non-isolated, the patient was subsequently excluded from the analysis.

At the time of data analysis (2009–2016) the study sample comprised of 619 ARM patients and their families. After exclusion of all syndromic patients (*n* = 292) 327 patients with isolated ARM (53%) remained. From these remaining 327 patients with isolated ARM data on first degree relatives were evaluated to identify multiple affected families.

## Results

### Multiplex ARM families with affected first degree relatives

#### Affected parents with affected children (parent-offspring families)

Within our cohort of 327 patients with isolated ARM we identified only eight patients from eight families who had in total 16 children with their healthy spouse (Table 1, Additional file 1: Figure S1). This low number of patients with children is due to the fact, that the majority of patients in our cohort are underage. Here, four men with isolated ARM fathered five affected and one healthy child. Four women with isolated ARM had five affected and five healthy children. Hence, the recurrence risk of ARM among 16 offspring is approximately one in two live births (10 of 16; 62%; 95%-CI: 35% - 85%). Noteworthy, in two of these nine families have been further affected family members (Table 1, Additional file 1: Figure S1): In family 1 another relative of the affected father (anal atresia) had unspecified ARM. The grandparents and the sister of the affected father of family 4 had unspecified ARM.

**Table 1:** Phenotypic description of parents with isolated ARM

Family	Affected parent	Phenotype of parent	Unaffected offspring	Affected offspring	Additionally affected family members
1	Father	NS	–	2 (male, perineal fistula; male perineal fistula)	One further relative in paternal family
2	Father	NS	1	1 (male, perineal fistula)	–
3	Father	Anal stenosis	–	1 (male, perineal fistula)	–
4	Father	NS	–	1 (male, perineal fistula)	Grandparents and the sister of the affected father had unspecified ARM
5	Mother	Anterior ectopy	–	2 (male, perineal fistula; female, anterior ectopy)	–
6	Mother	Perineal fistula	–	2 (female, perineal fistula; male, anal atresia without fistula and aganglionic blind-loop)	–
7	Mother	Vestibular fistula	3	1 (female, vestibular fistula)	–
8	Mother	Perineal fistula	2	–	–

*NS* not specified

#### Phenotypic description of ARM subphenotypes among parent-offspring families

Of the four fathers with isolated ARM one had anal atresia and another had a stenosis of the anal canal; the type of ARM in the other two fathers is unspecified. In their offspring we recorded five perineal fistula. Of the four mothers with isolated ARM two had a perineal fistula, another one had a vestibular fistula and one had an ectopic anus (Table 1, Additional file 1: Figure S1). One of the families has been published before (family 6) [6]. In their offspring we observed two perineal fistula, one vestibular fistula, one atresia without fistula (aganglionic blind-loop) and one ectopic anus.

#### Healthy parents with two affected children

In 226 families (69%) we found 459 siblings. In five of these families two siblings were affected with ARM (Table 2, Additional file 1: Figure S1). Hence, the recurrence risk of ARM among siblings is approximately one in 92 live births (5 of 459; 1.0%; 95%-CI: 0.3% - 2.3%). Affected siblings with affected parents have been excluded for this analysis, but included in the figures for vertical transmission.

**Table 2:** Phenotypic description of affected siblings with non-syndromic ARM from unaffected parents

Family	Unaffected offspring	Affected offspring	Additionally affected family members
9	–	2 (female, perineal fistula; female, perineal fistula)	–
10	–	2 (male, perineal fistula; female, cloacal malformation)	Maternal second cousin with not specified ARM
11	1	2 (male, perineal fistula; male, NS)	–
12	1	2 (female, perineal fistula; male, anal atresia without fistula)	–
13	1	2 (male, prostatic fistula; female, vestibular fistula)	–

#### Phenotypic description of ARM subphenotypes among families with affected siblings

Of the five families with two affected children (five female and five male) five children showed perineal fistula, one with a recto-urethral fistula, one had a cloacal malformation, one with an anal atresia and one unspecified ARM. Additionally, there have been three healthy siblings (families 11, 12 and 13). In family 10 a history of ARM with a maternal second cousin with unspecified ARM has been reported.

#### Recurrence risk in families with specific subphenotypes

Of the 197 patients with perineal or vestibular fistula 17 had at least one further family member affected with ARM (8.6%; 95%-CI: 5.1% - 13.5%), resulting in a 260-fold increase in risk to the general population. From 29 patients with a prostatic fistula only one had an affected family member (3.5%; 95%-CI: 0.1% - 17.8%), resulting in a 103-fold increased risk compared to the general population.

## Discussion

Data on recurrence risk for genetic counseling is sparse for ARM and is mostly limited to reports of familial occurrence. Reliable figures however will become increasingly important, since improved surgical techniques are able to maintain sexual function resulting in more offspring of patients with ARM. Here, we present data on the recurrence risk in a large cohort for isolated ARM and determine the recurrence risk in offspring of parents with ARM, which has not been investigated previously. The first report on the recurrence risk in families with ARM estimated the recurrence risk for siblings to be 1% [7]. Although this figure is based on unknown and unquoted reports, it is often used as a reference. In 2007 Falcone et al. identified in 2.4% of 1606 ARM patients an additional family member with a congenital malformation, which was an ARM in 1.4% [8]. Comparing the recurrence risk we observed in the studied cohort with the estimated prevalence in the general population of 1 in 3000 we see a 1500-fold increase in recurrence risk for offspring and a 32-fold increase if a sibling is affected. The recurrence risk of 62% (10 affected of 16 offspring in 8 families) indicates an autosomal dominant mode of inheritance. However, we have to emphasize that the estimation of recurrence risk is not precise due to the low number of cases. Not only larger population-based cohorts will be necessary to refine exact figures but also further genetic studies are needed to identify explicit genetic factors.

In spite of this impressive increased risk, we have to consider that the number of parents is still low in our cohort with mainly underage patients. This is due to the fact that pediatric surgeries could recruit only underage patients, and parents in our cohort were mainly recruited through the German self-help group. There may be an increased motivation for an affected parent with an affected child than an affected parent with healthy offspring to participate in a genetic study, hence selection bias cannot be excluded. On the other side we know that some parents with minor forms of anorectal malformation (anal stenosis or anus copertus) were not considered as affected, until they had an affected child. The marked difference in recurrence rates between offspring of affected parents on the one hand and siblings on the other hand raises further questions. First, our data indicates autosomal dominant mode of inheritance for offspring of affected parents. Second, we see a lower rate when observing recurrence risk of ARM in siblings. Third, a fraction of ARM in the latter group can be still explained by dominant alleles if they arise de novo. Fourth, since ARM is a disease reducing fecundity, we hypothesize that this gain of new mutant alleles would compensate for allele loss. Comparable mechanisms have been demonstrated for other diseases that compromise individual fecundity explaining this paradox [10].

Previous findings reported epidemiologic differences among the various types of anal anomalies suggesting different embryological or genetic origins [1]. Notably, Falcone et al. found patients with a perineal or vestibular fistula having an increased risk to have a relative with ARM. In those families ARM recurred in 3% of families, resulting in a 90-fold increase in risk compared with the general population (based on a frequency in the general population of 1 in 3000). In contrast, in patients with cloacal malformations or prostatic fistula they observed a significantly lower risk of recurrence among family members [8]. According to this data, we confirmed that there is a higher risk of recurrence in the presence of a perineal or vestibular fistula among first degree relatives. Unlike Falcone et al. we did not find a difference in sex ratios, namely a female predominance, in the group of ARM patients with at least one affected family member.

## Conclusion

Our data in a large cohort of isolated ARM indicates a higher recurrence risk of ARM than previously reported. Especially for offspring of affected patients with ARM we demonstrate a recurrence risk of approximately 62% suggesting an autosomal dominant mode of inheritance. However, research has yet only identified a minority of the monogenic factors.

## Additional file

Additional file 1: Figure S1. Pedigrees of 13 families with ARM. Eight parent-offspring families with 16 children and five families with healthy parents with two affected children (PPTX 80 kb)
