# Supplementary material for: Epidemiologic analysis of families with isolated anorectal malformations suggests high prevalence of autosomal dominant inheritance
Source: Orphanet J Rare Dis. 2017 Dec 13;12:180. doi: 10.1186/s13023-017-0729-7 (PMC5729416; doi:10.1186/s13023-017-0729-7)
Supplement: Additional file 1: Figure S1. — Pedigrees of 13 families with ARM. Eight parent-offspring families with 16 children and five families with healthy parents with two affected children (PPTX 80 kb) [file 13023_2017_729_MOESM1_ESM.pptx]

## Slide 1
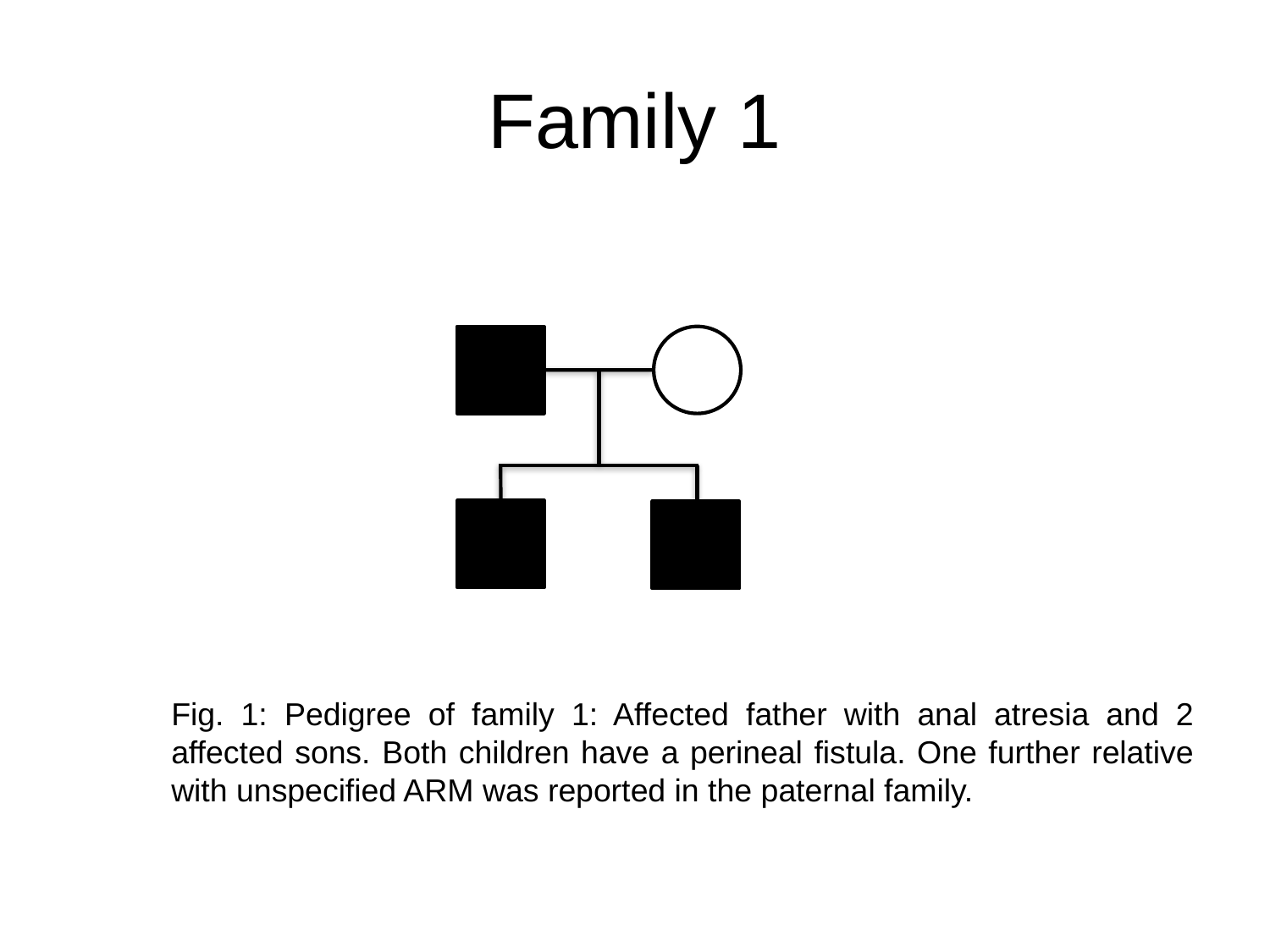

Family 1
Fig. 1: Pedigree of family 1: Affected father with anal atresia and 2 affected sons. Both children have a perineal fistula. One further relative with unspecified ARM was reported in the paternal family.

## Slide 2
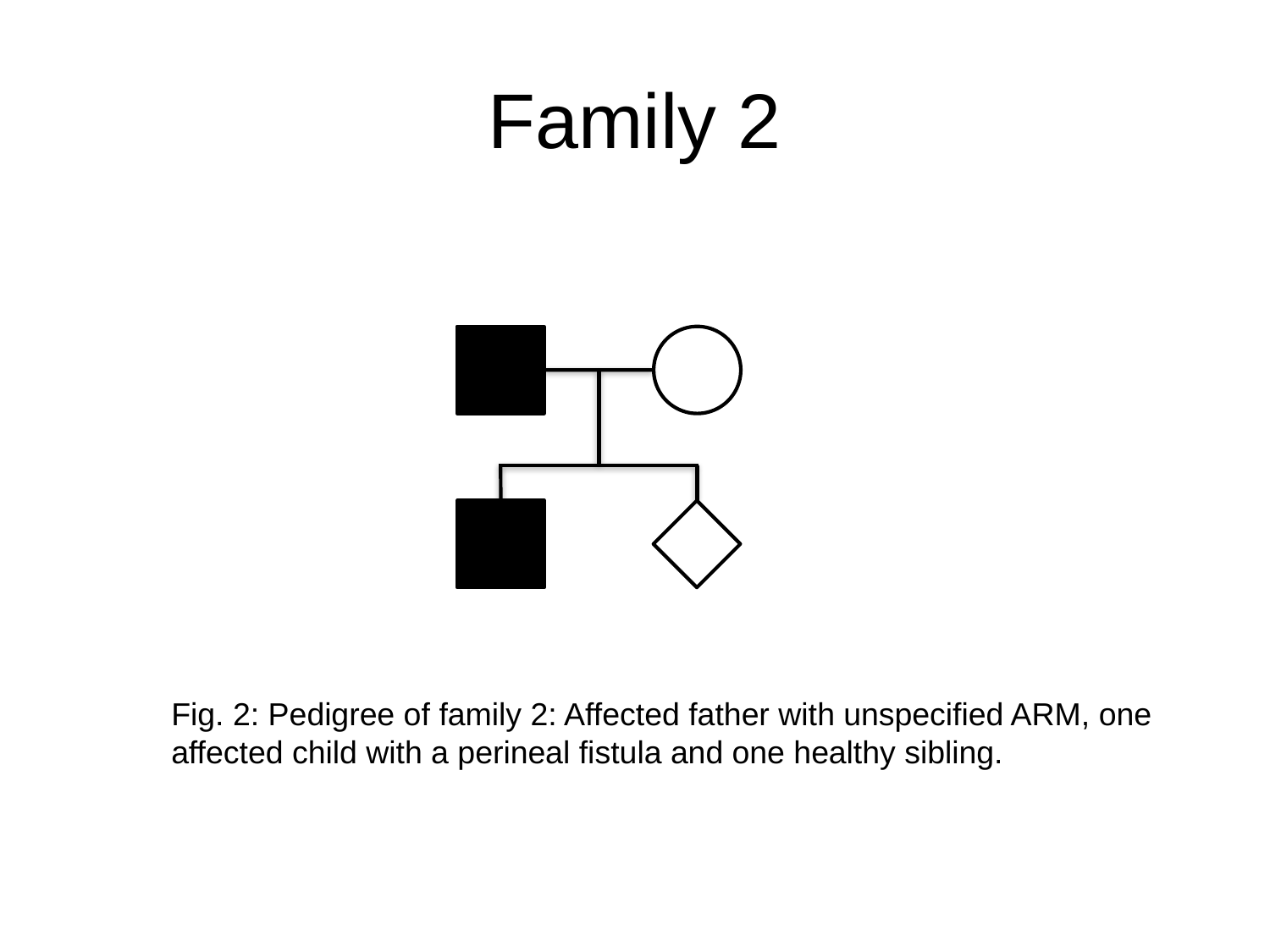

Family 2
Fig. 2: Pedigree of family 2: Affected father with unspecified ARM, one affected child with a perineal fistula and one healthy sibling.

## Slide 3
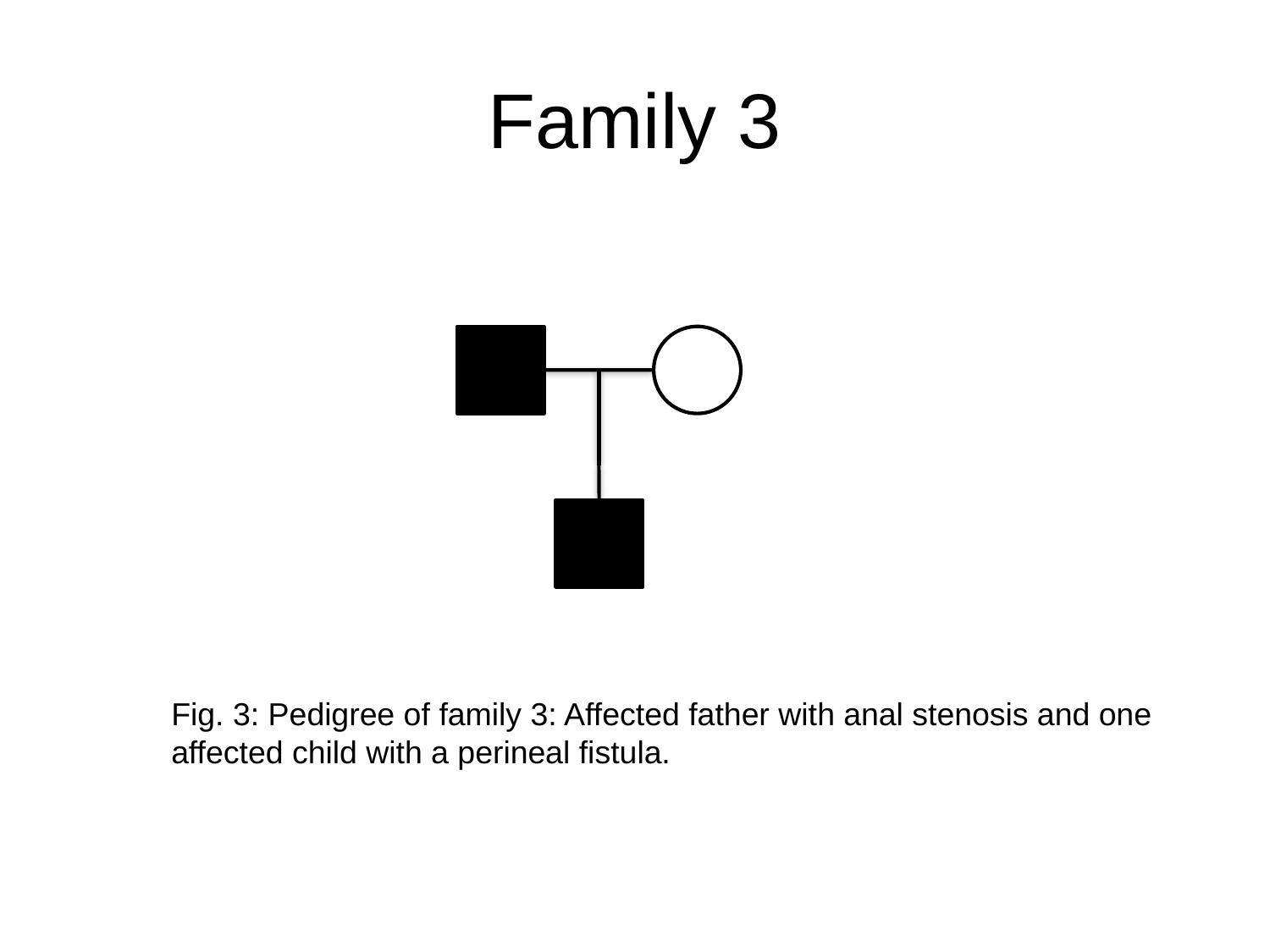

Family 3
Fig. 3: Pedigree of family 3: Affected father with anal stenosis and one affected child with a perineal fistula.

## Slide 4
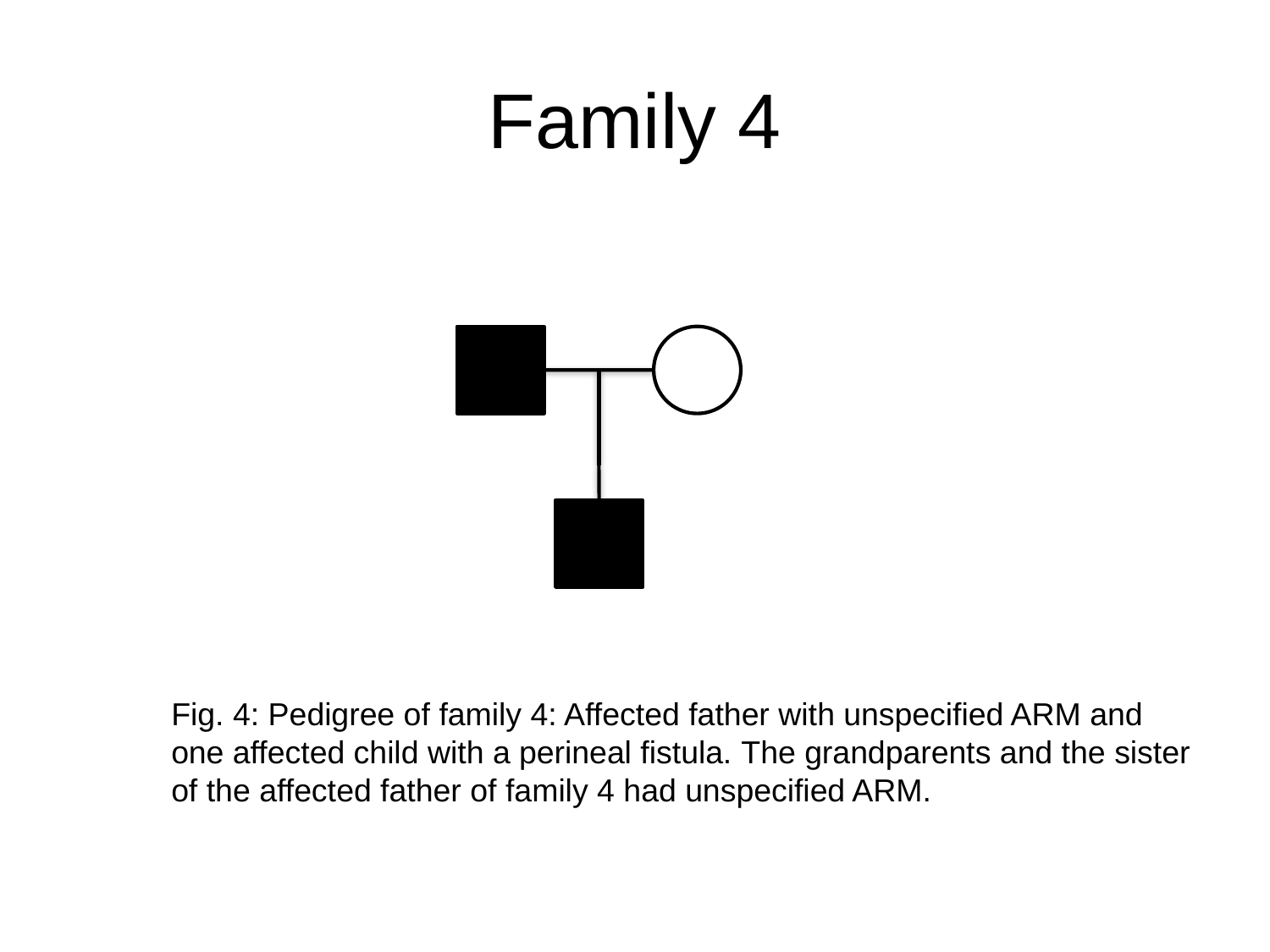

Family 4
Fig. 4: Pedigree of family 4: Affected father with unspecified ARM and one affected child with a perineal fistula. The grandparents and the sister of the affected father of family 4 had unspecified ARM.

## Slide 5
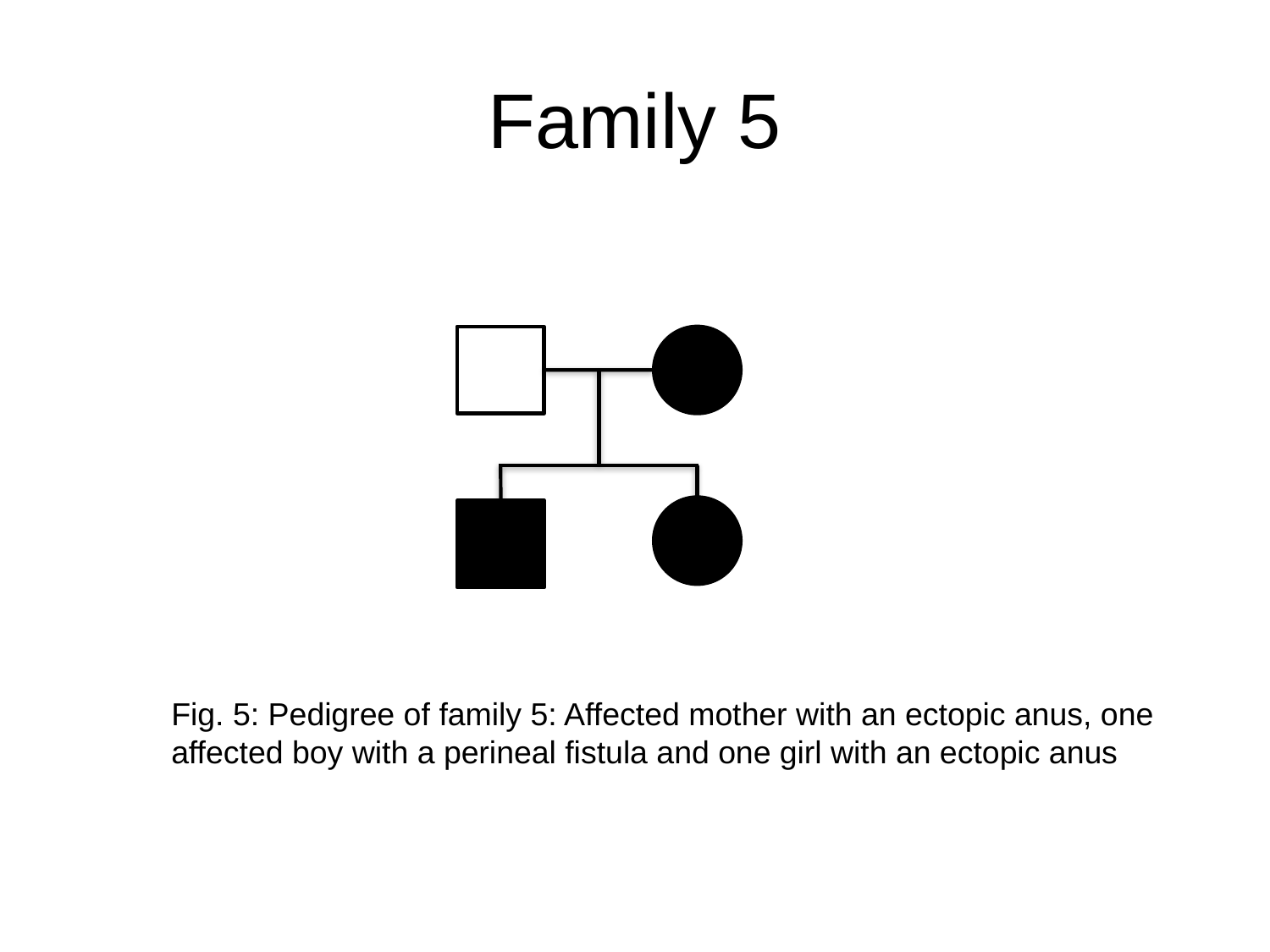

Family 5
Fig. 5: Pedigree of family 5: Affected mother with an ectopic anus, one affected boy with a perineal fistula and one girl with an ectopic anus

## Slide 6
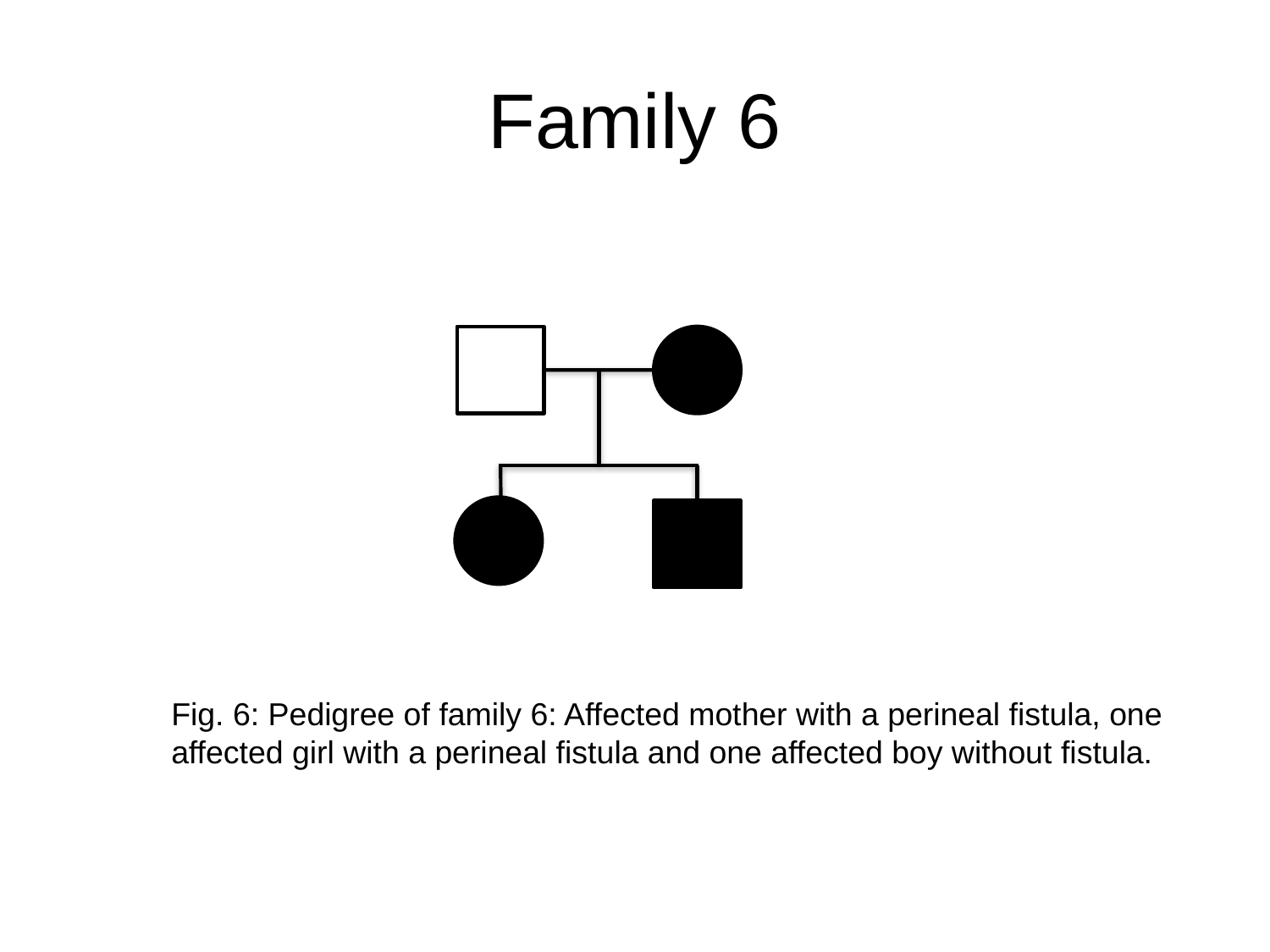

Family 6
Fig. 6: Pedigree of family 6: Affected mother with a perineal fistula, one affected girl with a perineal fistula and one affected boy without fistula.

## Slide 7
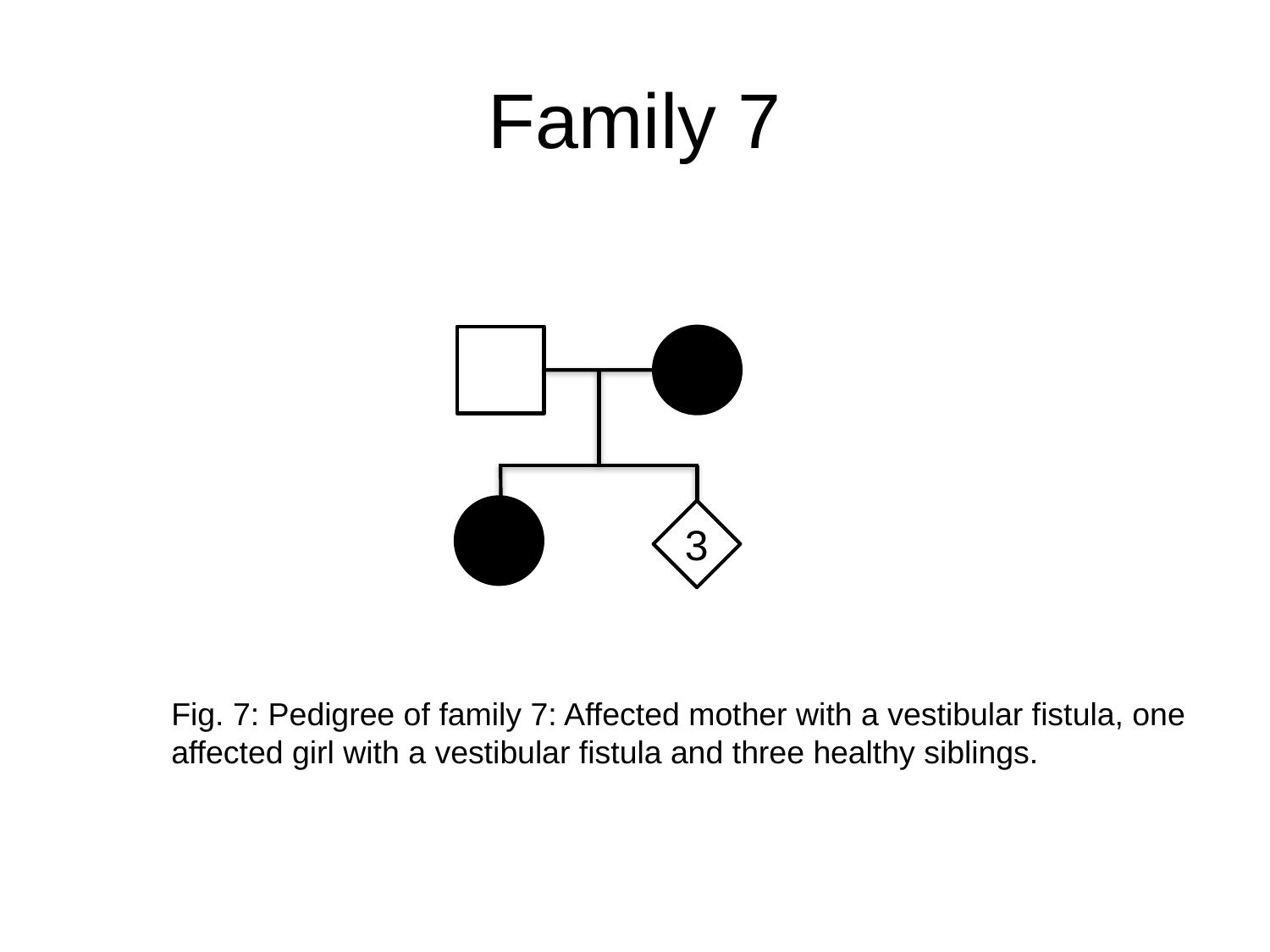

Family 7
3
Fig. 7: Pedigree of family 7: Affected mother with a vestibular fistula, one affected girl with a vestibular fistula and three healthy siblings.

## Slide 8
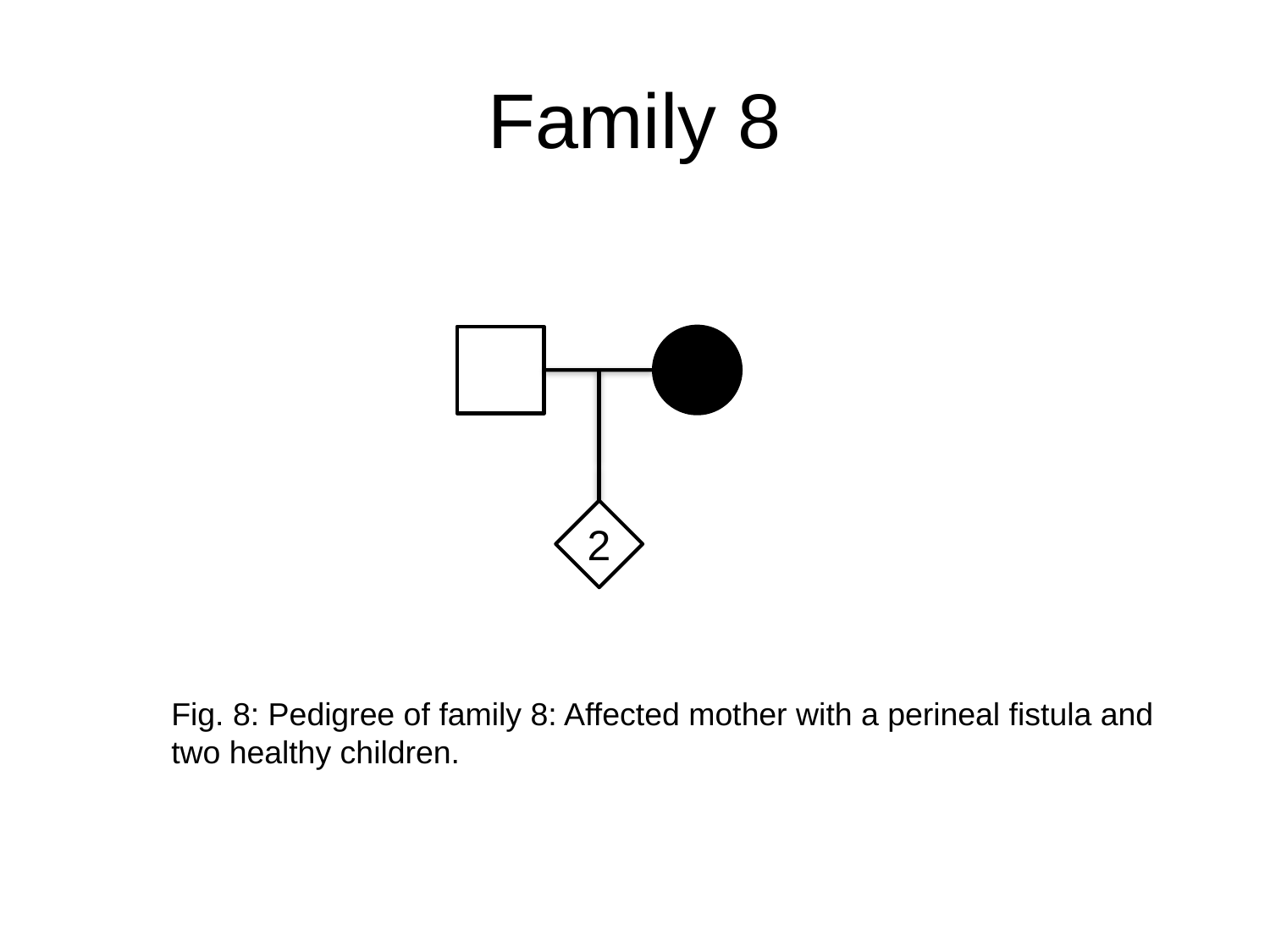

Family 8
2
Fig. 8: Pedigree of family 8: Affected mother with a perineal fistula and two healthy children.

## Slide 9
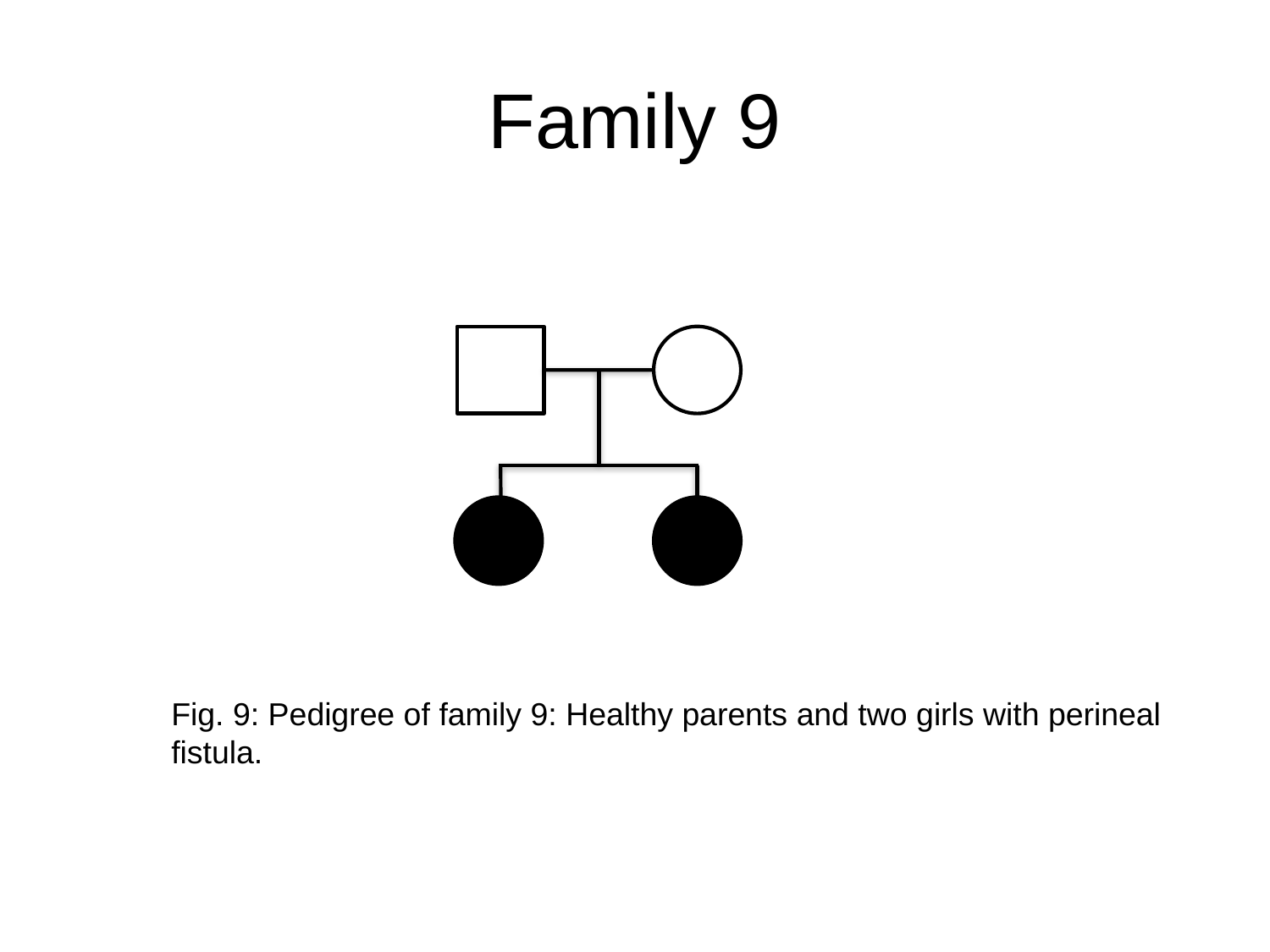

Family 9
Fig. 9: Pedigree of family 9: Healthy parents and two girls with perineal fistula.

## Slide 10
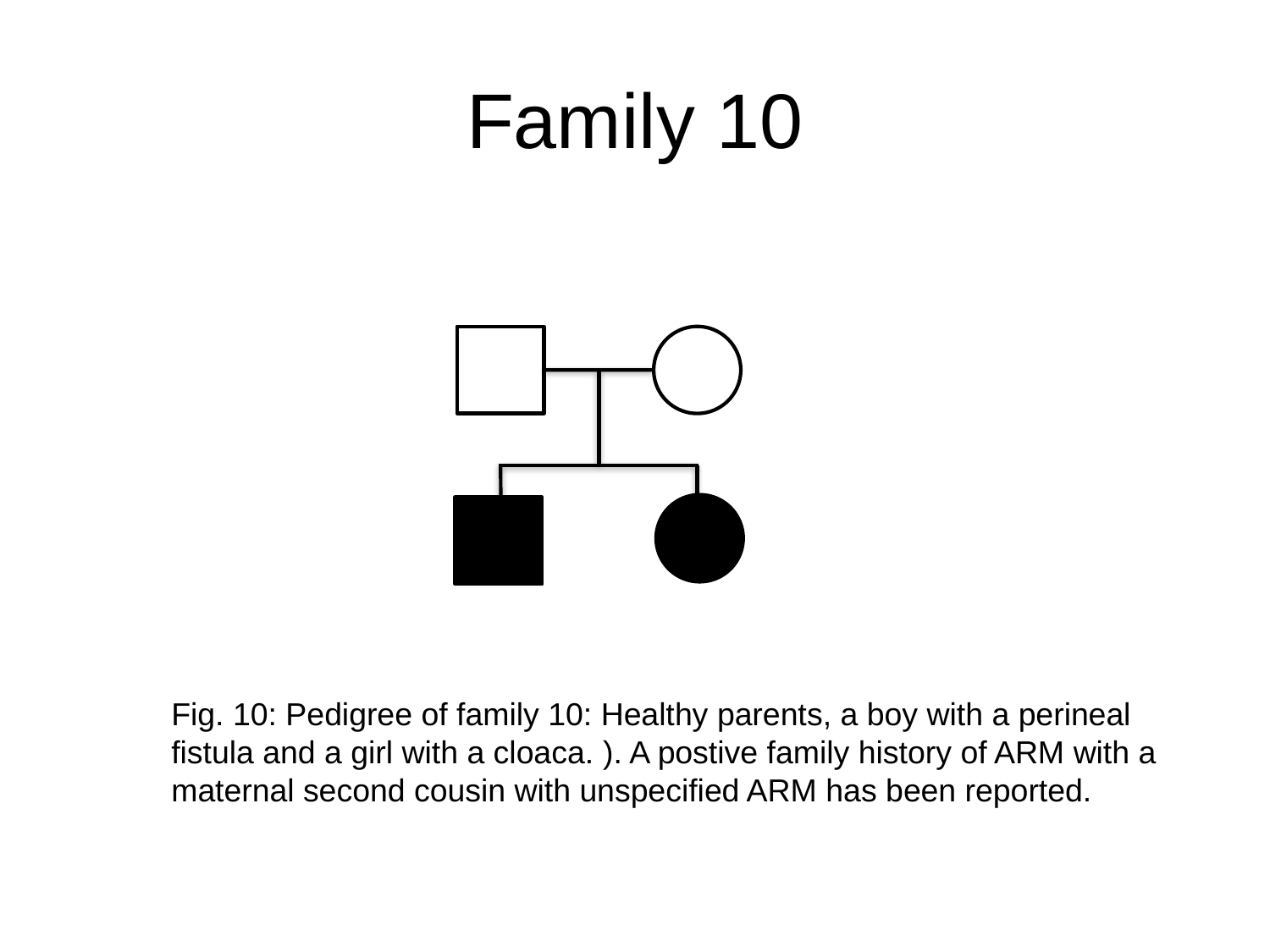

Family 10
Fig. 10: Pedigree of family 10: Healthy parents, a boy with a perineal fistula and a girl with a cloaca. ). A postive family history of ARM with a maternal second cousin with unspecified ARM has been reported.

## Slide 11
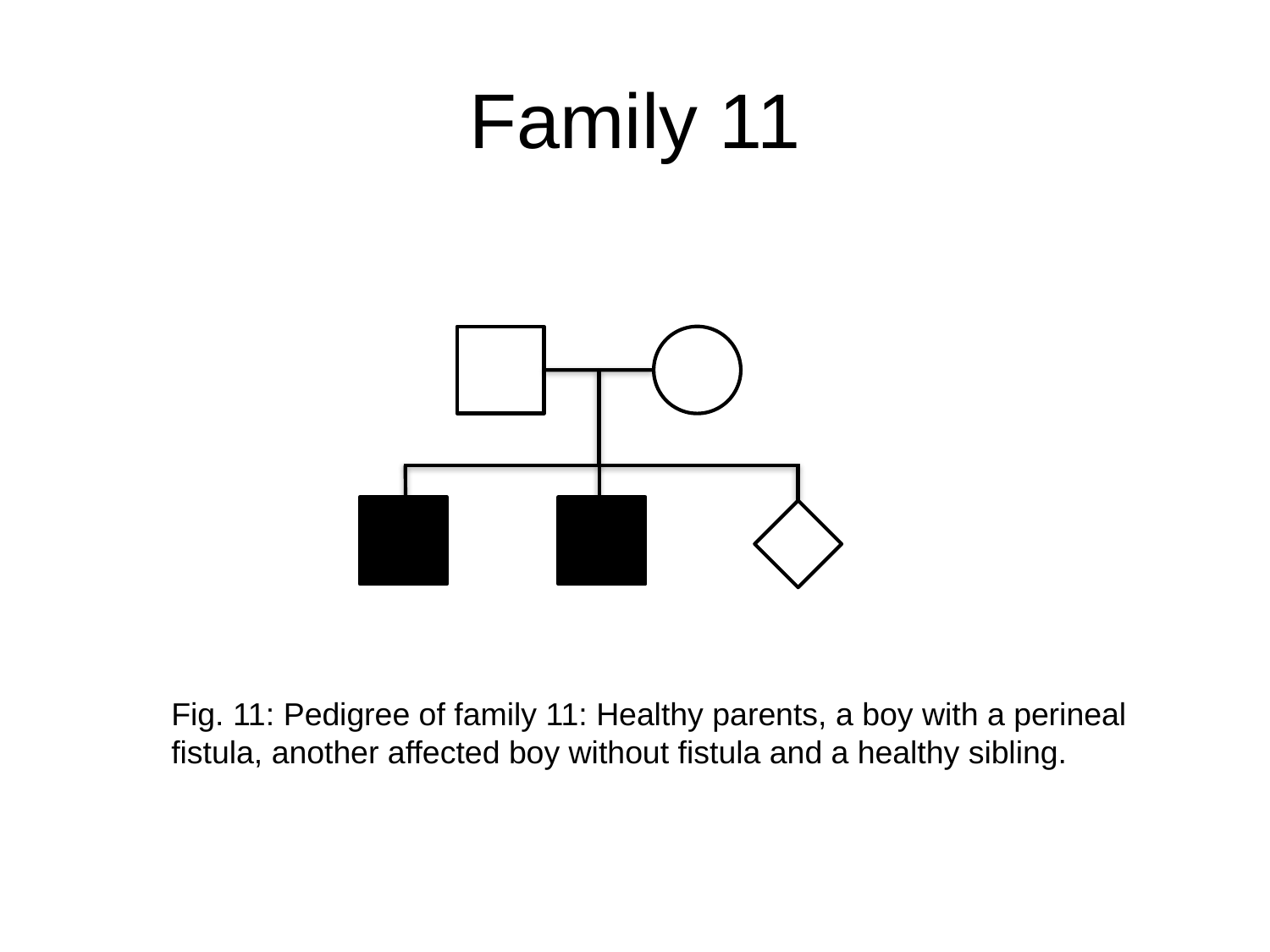

Family 11
Fig. 11: Pedigree of family 11: Healthy parents, a boy with a perineal fistula, another affected boy without fistula and a healthy sibling.

## Slide 12
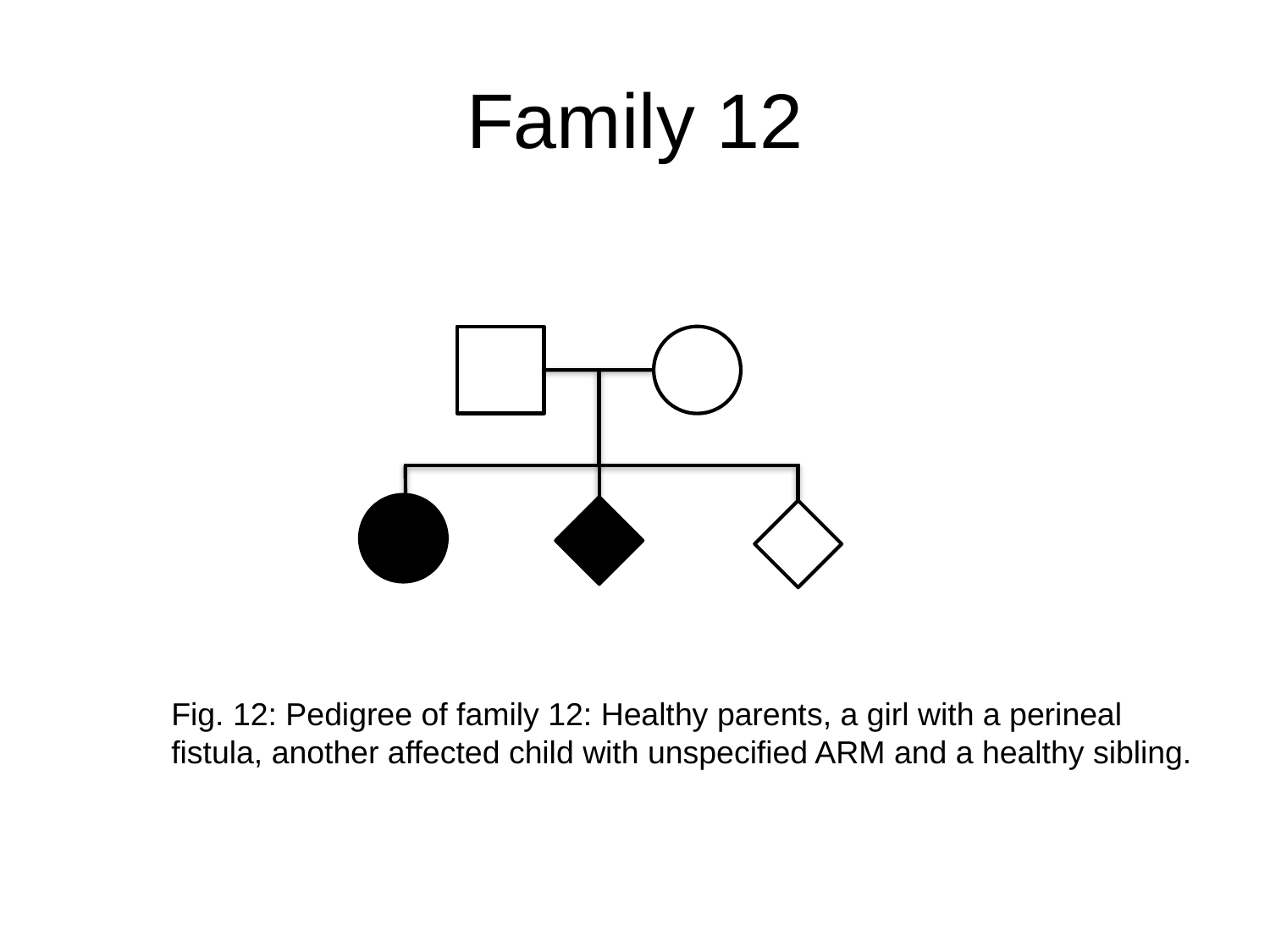

Family 12
Fig. 12: Pedigree of family 12: Healthy parents, a girl with a perineal fistula, another affected child with unspecified ARM and a healthy sibling.

## Slide 13
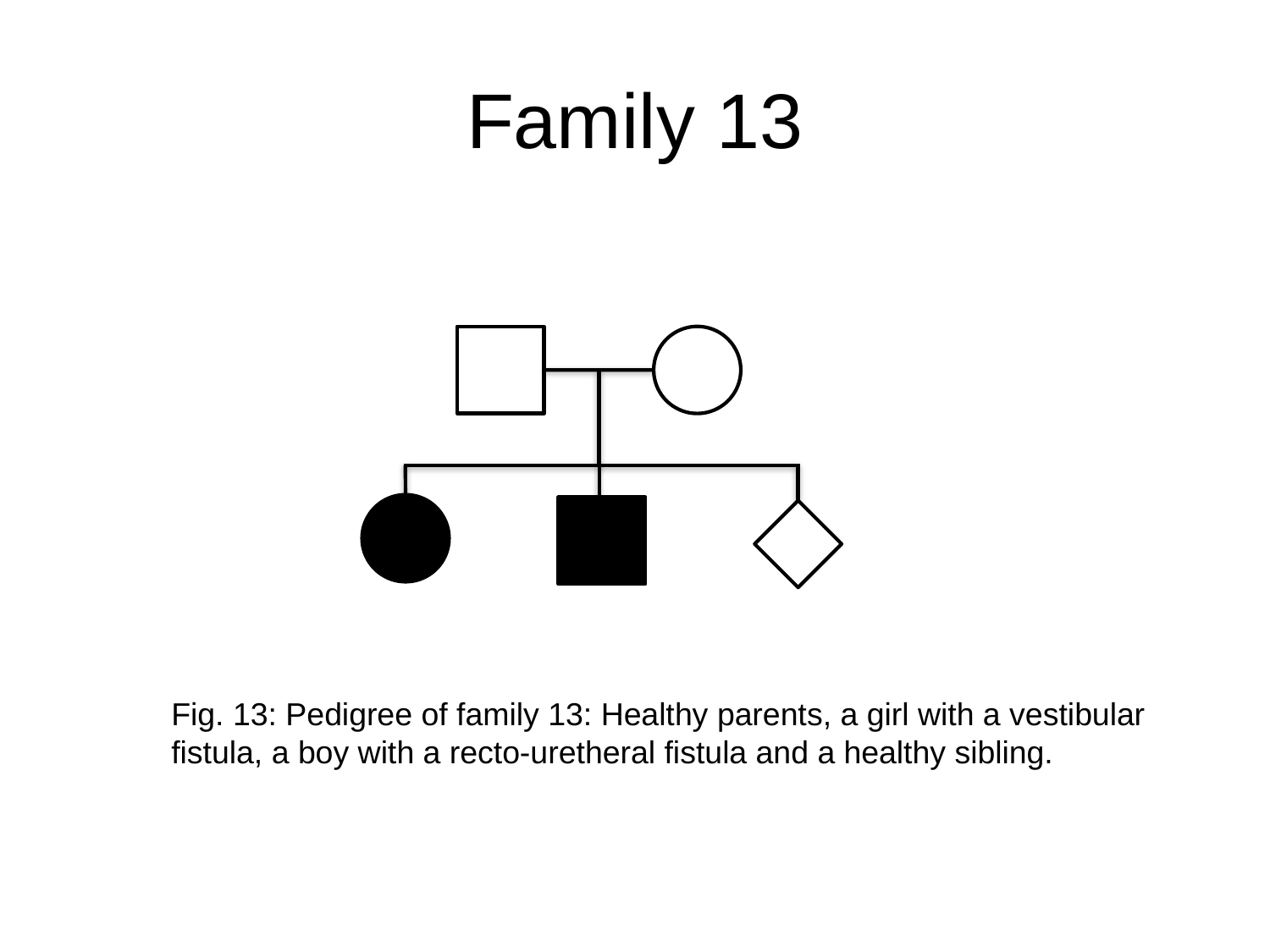

Family 13
Fig. 13: Pedigree of family 13: Healthy parents, a girl with a vestibular fistula, a boy with a recto-uretheral fistula and a healthy sibling.
